# Prevalence of Histologically Positive Helicobacter pylori Infection Among Patients Who Underwent Upper GI Endoscopy at the University Hospital Limerick in 2023

**DOI:** 10.7759/cureus.94522

**Published:** 2025-10-14

**Authors:** Adnan Abdalla, Swraj Singla, Aakash Lakhani, Nafisa Hemedy, Waleed Ahmed, Ahmed Aydrose, Musab Suliman, Mutwaly Haron, Pardeep Maheshwari

**Affiliations:** 1 Internal Medicine, University Hospital Limerick, Limerick, IRL; 2 General Surgery, University Hospital Limerick, Limerick, IRL

**Keywords:** esophagogastroduodenoscopy, gastritis, gi endoscopy, helicobacter, prevalence

## Abstract

Background: Data on *Helicobacter pylori* *(H. pylori) *prevalence in Ireland are limited. This study aimed to determine the prevalence of histologically positive *H. pylori *infection among patients undergoing upper gastrointestinal endoscopy at University Hospital Limerick (UHL), Ireland.

Methods: A cross-sectional study was conducted at UHL between January and December 2023. Adult patients who underwent upper gastrointestinal endoscopy with gastric biopsy were included. Histopathology was the primary diagnostic method, with the Campylobacter-like organism (CLO) rapid urease test in a subset. Demographics, clinical indications, and endoscopic findings were analyzed using descriptive statistics and the Chi-square test.

Results: Of 1023 patients who underwent endoscopy with gastric biopsies, 775 were tested histologically, and 103 (13.29%) were positive for *H. pylori*. Prevalence increased with age, peaking in the 46-55-year group. No significant gender difference was observed (13.9% males vs. 12.6% females, p = 0.295). Gastritis (59.2%) was the predominant endoscopic finding, and CLO test results correlated significantly with histopathology (p < 0.001).

Conclusion: The 13.2% prevalence aligns with rates in other developed settings but remains below global averages. The detailed geographical breakdown, particularly the predominance of cases from Co. Limerick, provides valuable insight into the local epidemiological landscape. The results highlight regional distribution patterns in Ireland and support the continued combined use of histology and rapid urease testing for accurate diagnosis.

## Introduction

*Helicobacter pylori* (*H. pylori*) is one of the most prevalent bacterial infections worldwide, colonizing nearly half of the global population [1]. It is recognized as a major cause of chronic gastritis, peptic ulcer disease, and gastric cancer [2,3]. The International Agency for Research on Cancer has classified *H. pylori* as a Group I carcinogen due to its causal association with gastric adenocarcinoma [4]. Globally, prevalence rates vary markedly, with rates exceeding 80% in developing countries and 20-30% in most developed regions [1, 5-8].

Hospital-based prevalence data from patients undergoing endoscopy are particularly important as they represent a different population subset with active gastrointestinal symptoms, and such data provide useful information for clinical decision-making and comparison with international endoscopic series. Endoscopies provide an in-depth microscopic assessment of GI mucosa compared to any other modality by allowing for the collection of biopsies that can be used for invasive diagnostic tests like the rapid urease test and histopathological examination [9-10].

Despite this global significance and clinical importance, data from Ireland remain limited. Understanding local prevalence in Ireland is essential for clinical management, monitoring temporal trends, and public health planning. The prevalence data will provide valuable epidemiological data for the Irish healthcare system. Therefore, this study aimed to determine the prevalence of histologically positive *H. pylori* infection among patients undergoing upper gastrointestinal endoscopy at University Hospital Limerick (UHL) in 2023.

## Materials and methods

This was a cross-sectional, hospital-based study conducted in the Department of Gastroenterology at the UHL, Ireland, over a 12-month period from January to December 2023. UHL is the main public referral hospital for the Limerick region and serves as a central hub for emergency and elective services, including endoscopic procedures.

The study population comprised all adult patients (aged ≥18 years) who underwent upper gastrointestinal (GI) endoscopy with gastric biopsy at UHL during the study period. Patients were included consecutively to minimize selection bias. The inclusion criterion was a completed upper GI endoscopy with at least one gastric biopsy sample taken for histopathological examination. Exclusion criteria included: (1) patients who underwent endoscopy without biopsy, (2) patients who had duodenal-only, esophageal-only, or esophagogastric junction-only biopsies, and (3) patients with recent antibiotic or *H. pylori* eradication therapy.

Data were collected using a structured questionnaire designed specifically for the study. The questionnaire captured a range of variables, including demographics, clinical presentation, endoscopic findings as recorded by the endoscopist during the procedure, and histopathological findings, with a focus on the detection of *H. pylori*. Histopathological examination of biopsy specimens was the primary diagnostic method for *H. pylori* detection. All tissue sections were reviewed by an experienced pathologist to determine the presence or absence of *H. pylori*.

In addition, a subset of patients also underwent rapid urease testing using the Campylobacter-like organism (CLO) test (Delta West Pty Ltd., Bentley, Australia), which was performed at the time of endoscopy and biopsy to assess the correlation between CLO results and histopathology results.

A detailed geographical distribution analysis was performed using patient address data to identify regional patterns of *H. pylori* positivity within County Limerick (where the UHL is located). County-level and subdivision-level mapping included Limerick City and the following internal administrative subdivisions: Clanwilliam, Glenquin, Pubblebrien, Coshma, Shanid, Coonagh, Coshlea, Connello Lower, Connello Upper, and Pallaskenry.

Statistical analysis was performed using SPSS Version 20 (SPSS Inc., Chicago, IL, USA). Descriptive statistics were used to summarize the sample characteristics, including frequencies, percentages, and cross-tabulations. The Chi-square test was used to examine associations between categorical variables, such as the relationship between *H. pylori* status and demographic or clinical features. A p-value of less than 0.05 was considered statistically significant. Ethical approval was obtained from the UHL ethics committee, and study data were used for research purposes only, and confidentiality was ensured for all the participants.

## Results

**Figure 1:**
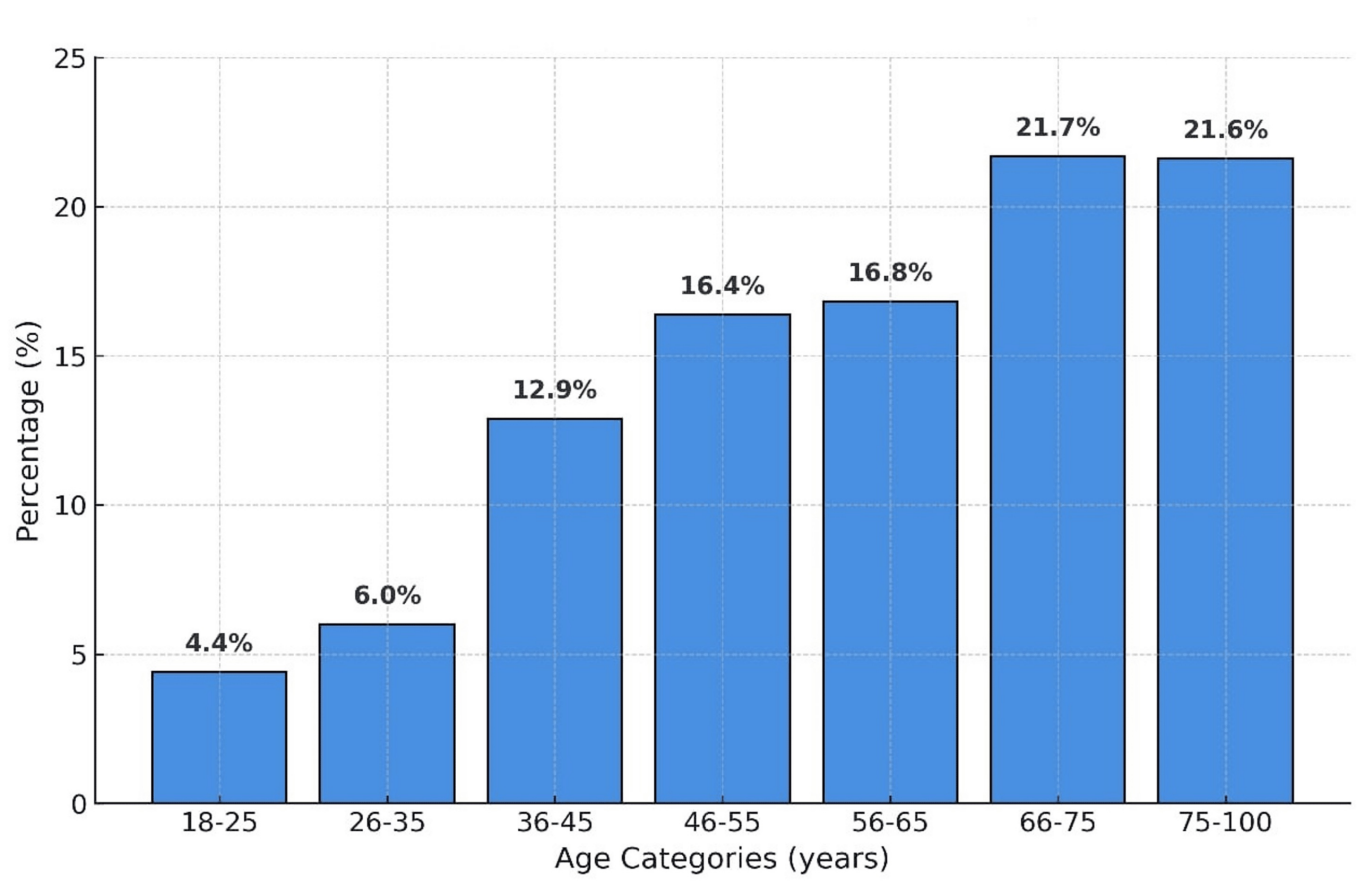
Age groups of the participants.

Of 1023 endoscopy procedures performed during the study period, 775 patients met the inclusion criteria and were tested histologically. The majority of the participants were from the older age groups (Figure 1). The overall prevalence of *H. pylori* infection was 13.2% (n = 103). Infection was slightly more common in males (13.9%) compared with females (12.6%), but the difference was not statistically significant (p = 0.295). Among those who tested positive, the 46-55 and 36-45 age groups contributed the most cases (21.4% and 20.4% respectively), while the youngest and oldest groups contributed the fewest cases. Regarding the CLO test, 345 patients had the test during OGD. Among the 25 patients who had positive CLO test, 18 patients had positive histopathology for *H. pylori*, while 286 out of the 320 who had negative CLO test also had negative histopathology for *H. pylori*. The results demonstrated a strong and significant correlation with histopathology (p < 0.001), confirming diagnostic reliability (p< 0.001) (Table 1).

**Table 1:** Detection of Helicobacter pylori infection by histopathology and diagnostic method correlation *OGD: Esophago-gastro-duodenoscopy

Parameter	Category	Positive	Negative	X^2^	p-value
Association with gender	Male	54	333	0.295	.587
	Female	49	339		
Association with age groups	18-25	7	32	17.92	.006
	26-35	6	47		
	36-45	21	94		
	46-55	22	124		
	56-65	19	131		
	66-75	19	174		
	75-100	9	183		
Association with CLO Test during OGD (n=345)	CLO +ve	18	7	68.32	
	CLO -ve	34	286		

Dyspepsia was the most frequent clinical indication, reported in 57.3% of *H. pylori*-positive patients, followed by gastritis (37.9%) and abdominal pain (32.0%). Less common symptoms included dysphagia (9.7%), anemia (8.7%), and weight loss (5.8%). Endoscopically, gastritis predominated across clinical and endoscopic findings, while ulcers and vascular abnormalities were uncommon. In the duodenum, the majority of patients (73.8%) had normal mucosa; however, duodenitis was observed in 18.4% and duodenal ulcer in 2.9%. Histopathology revealed active gastritis in 92.2% of infected patients, while 7.8% showed inactive gastritis. Clinical presentations and endoscopic findings are summarized in Table 2.

**Table 2:** Clinical presentations and endoscopic findings in Helicobacter pylori-positive patients (n=103)

Parameter	Category	N (positive)	%
Clinical Indications	Dyspepsia	59	57.3
	Gastritis	39	37.9
	Abdominal pain	33	32.0
	Dysphagia	10	9.7
	Anemia	9	8.7
	Weight loss	6	5.8
Gastric Endoscopic Findings	Gastritis	61	59.2
	Normal appearance	31	30.1
	Gastric ulcer	7	6.8
	Polyp	2	1.9
	Gastric varices	1	1.0
	Portal hypertensive gastropathy	1	1.0
Duodenal Endoscopic Findings	Normal appearance	76	73.8
	Duodenitis	19	18.4
	Duodenal ulcer	3	2.9
	Polyp	2	1.9
	Diverticulum	2	1.9
	Thick folds	1	1.0
Esophageal Endoscopic Findings	Normal	55	53.4
	Esophagitis	22	21.3
	Hiatus Hernia	19	18.4
	Barrett’s Esophagus	3	2.9
	Candida	1	1.0
	Lax sphincter	1	1.0
	Malignancy	1	1.0
	Esophageal Varices	1	1.0
Microscopic findings of gastric biopsies	Active gastritis	95	92.2
	Inactive gastritis	8	7.8

The geographical distribution of cases is presented in Table 3. Geographical analysis showed that most positive cases were from County Limerick (61.2%), with a notable concentration in Limerick City (25.4%). Smaller proportions were recorded from County Clare (18.4%) and County Tipperary (14.6%). Within County Limerick, the highest burden was recorded in Limerick City (25.4%), Clanwilliam (15.9%), and Glenquin(12.7%). Smaller numbers of cases were distributed across other rural districts (Table 3).

**Table 3:** Geographical distribution of Helicobacter pylori positive cases *Other counties: Wexford, Mayo, Offaly, Kildare, Cork, Wicklow (1 case each)

Region	Category	N (positive)	%
All counties	County Limerick	63	61.2
	County Clare	19	18.4
	County Tipperary	15	14.6
	Other counties*	6	5.8
Within County Limerick (n=63)	Limerick City	16	25.4
	Clanwilliam	10	15.9
	Glenquin	8	12.7
	Pubblebrien	7	11.1
	Coshma	5	7.9
	Shanid	4	6.3
	Coonagh	4	6.3
	Coshlea	3	4.8
	Connello Lower	3	4.8
	Connello Upper	2	3.2
	Pallaskenry	1	1.6

## Discussion

Diseases of the upper gastrointestinal tract contribute substantially to global morbidity and mortality, and histopathological evaluation of endoscopic biopsies remains central to accurate diagnosis and management. This study provides updated data on the prevalence of *H. pylori* infection among patients undergoing upper gastrointestinal endoscopy at a tertiary hospital in Ireland. The histologically confirmed prevalence of 13.2% is relatively low compared to global figures but consistent with trends in other developed countries. A meta-analysis by Hooi et al. reported a worldwide prevalence of nearly 50%, with the highest rates in Africa and South America, while Western Europe demonstrated markedly lower prevalence levels [1]. Our findings align with data from England and Wales, where McNulty et al. [11] reported a prevalence rate of 11% among endoscopy patients, and with data from Canada, where Willems et al reported a prevalence rate of around 13% [12]. In Europe, prevalence demonstrates a north-south gradient, with the lowest rates in Scandinavia (near 20%) and higher rates approaching 40% in southern regions. These similarities suggest that Ireland is following the same trend observed across developed regions.

Several factors may explain the relatively low prevalence observed in this study, including improved hygiene, higher socioeconomic status, and effective eradication programs. These factors have all been implicated in reducing transmission of *H. pylori* in high-income countries [5-6]. Furthermore, early detection and eradication strategies implemented in symptomatic patients may have contributed to the decline [13]. This is in contrast to developing countries, where persistent overcrowding, lower socioeconomic conditions, and limited access to eradication therapy maintain high prevalence levels [7]. The lack of age and gender difference observed in this study is consistent with the majority of published literature [9]. Some studies from Asia have reported slightly higher prevalence in men, potentially linked to lifestyle and occupational exposures, but such differences appear less relevant in European settings [6,8].

Geographically, the concentration of cases in County Limerick (61.2%) reflects the hospital’s catchment area, though the distribution across multiple counties suggests regional accessibility to specialized care. The identification of Limerick City as contributing 25.4% of county cases may indicate urban-rural differences in infection patterns or healthcare-seeking behavior. County Clare contributed 18.4% of cases despite being outside UHL's primary catchment area, suggesting either referral patterns from Clare to UHL or potentially higher *H. pylori* prevalence in this region, which warrants further investigation.

From a clinical perspective, dyspepsia was the leading indication for endoscopy among *H. pylori*-positive patients, which aligns with its well-established role in dyspepsia [12-13]. Endoscopic findings of gastritis were also common, reflecting the pathogen’s characteristic pathological changes. The absence of more severe outcomes such as gastric ulcers or malignancy in a significant proportion of infected patients highlights the heterogeneous clinical manifestations of H. pylori. This reinforces the importance of targeted rather than universal testing, consistent with the Maastricht V/Florence Consensus recommendations [13].

An additional finding of this study was the significant correlation between CLO test results and histological findings. Although histology remains the gold standard, rapid urease testing is inexpensive, minimally invasive, and provides immediate results [14]. Previous studies have demonstrated high concordance between CLO and histology for detecting *H. pylori*, supporting their complementary use in clinical settings [14-15]. The observed correlation suggests that, in appropriate clinical settings, the CLO test remains a reliable adjunct diagnostic tool.

The findings of this study have limitations that should be acknowledged. The study was single-center and hospital-based, which may not accurately reflect community prevalence. Patients undergoing endoscopy are typically symptomatic, and this selection bias may overestimate true prevalence in the general population. In addition, potentially relevant variables such as socioeconomic status, smoking, alcohol intake, and prior antibiotic exposure were not assessed. Finally, antimicrobial resistance patterns were not explored, which limits interpretation for treatment policy. Despite these limitations, this study provides valuable epidemiological data from Ireland, where the literature remains limited. The relatively low prevalence highlights the success of public health and clinical strategies in reducing infection rates. However, it also highlights the importance of caution, as *H. pylori* remains a key risk factor for peptic ulcer disease and gastric cancer in a minority of infected individuals. Overall, these results contribute to understanding *H. pylori* trends in Ireland and highlight the need for continued epidemiologic monitoring.

## Conclusions

The prevalence of histologically confirmed *H. pylori* infection at UHL was 13.2%, comparable to other developed settings but lower than global averages. The detailed geographical and clinical characterization provides valuable baseline data for Irish healthcare planning and supports evidence-based approaches to *H. pylori* diagnosis and management. This study provides valuable baseline data for Ireland, reinforcing the complementary diagnostic role of histology and CLO testing. Continued surveillance and population-based research are warranted to better define national prevalence and associated risk factors.

These findings contribute to the understanding of *H. pylori* epidemiology in Ireland and have important implications for targeted clinical management and public health strategies. These findings emphasize the need for continued surveillance in Ireland and support the combined use of histology and CLO testing for accurate diagnosis. Broader, population-based studies are recommended to determine the true national prevalence and risk factors.
